# Higher Adenoma Detection Rates with Endocuff-Assisted Colonoscopy – A Randomized Controlled Multicenter Trial

**DOI:** 10.1371/journal.pone.0114267

**Published:** 2014-12-03

**Authors:** Martin Floer, Erwin Biecker, Rüdiger Fitzlaff, Hermann Röming, Detlev Ameis, Achim Heinecke, Steffen Kunsch, Volker Ellenrieder, Philipp Ströbel, Michael Schepke, Tobias Meister

**Affiliations:** 1 Departments of Gastroenterology, HELIOS Albert-Schweitzer-Hospital Northeim, Northeim, Germany; 2 HELIOS Medical Center Siegburg, Siegburg, Germany; 3 HELIOS St. Marienberg Hospital Helmstedt, Helmstedt, Germany; 4 Institute of Biostatistics and Clinical Research, University of Münster, Münster, Germany; 5 Department of Gastroenterology, Zollernalb Medical Center, Bailingen, Germany; 6 Department of Gastroenterology II, University Medical Center Göttingen, Göttingen, Germany; 7 Institute of Pathology, University Medical Center Göttingen, Göttingen, Germany; University Hospital Llandough, United Kingdom

## Abstract

**Objectives:**

The Endocuff is a device mounted on the tip of the colonoscope to help flatten the colonic folds during withdrawal. This study aimed to compare the adenoma detection rates between Endocuff-assisted (EC) colonoscopy and standard colonoscopy (SC).

**Methods:**

This randomized prospective multicenter trial was conducted at four academic endoscopy units in Germany. Participants: 500 patients (235 males, median age 64[IQR 54–73]) for colon adenoma detection purposes were included in the study. All patients were either allocated to EC or SC. The primary outcome measure was the determination of the adenoma detection rates (ADR).

**Results:**

The ADR significantly increased with the use of the Endocuff compared to standard colonoscopy (35.4%[95% confidence interval{CI} 29–41%] vs. 20.7%[95%CI 15–26%], p<0.0001). Significantly more sessile polyps were detected by EC. Overall procedure time and withdrawal time did not differ. Caecal and ileum intubation rates were similar. No major adverse events occurred in both groups. In multivariate analysis, age (odds ratio [OR] 1.03; 95%[CI] 1.01–1.05), male sex (OR 1.74; 95%CI 1.10–2.73), withdrawal time (OR 1.16; 95%CI 1.05–1.30), procedure time (OR 1.07; 95%CI 1.04–1.10), colon cleanliness (OR 0.60; 95%CI 0.39–0.94) and use of Endocuff (OR 2.09; 95%CI 1.34–3.27) were independent predictors of adenoma detection rates.

**Conclusions:**

EC increases the adenoma detection rate by 14.7%(95%CI 6.9–22.5%). EC is safe, effective, easy to handle and might reduce colorectal interval carcinomas.

**Trial Registration:**

ClinicalTrials.gov NCT02034929.

## Introduction

Colorectal cancer derived from precancerous colonic lesions is one of the most frequently occurring tumors in the industrialized world. In a recently published study by Nishihara et al., it could be shown that endoscopic removal of polyps was associated with reduced incidences of colorectal cancer mortality [1]. The detection and removal of adenomas during colonoscopy is crucial to colon cancer prevention, since most types of colorectal cancer follow the adenoma-carcinoma sequence within 10 to 15 years (interval carcinoma). The adenoma detection rate (ADR), that is the proportion of colonoscopies during which at least one adenoma can be detected, is the most important factor for the prevention of colorectal interval carcinoma. In 2010, Kaminski et al. analyzed the database records of 50148 subjects from the National Colorectal Cancer Screening Program in Poland. In that study, the adenoma detection rate was shown to be an independent predictor of the risk of interval colorectal cancer after screening colonoscopy [2]. Several devices and techniques used during colonoscopy have been analyzed for improving the ADR [3]–[6]. Since 2012, a new endoscopic cuff (Endocuff) has been made available. Tsiamoulos and Saunders [7] first published their experience with the Endocuff for complex polyp resection procedures in the sigmoid colon in a small case series. In a recently published small feasibility study by Lenze at al., good procedural success rates for caecal intubation and procedure time, as well as a promising adenoma detection rate have been shown [8]. At present, only one prospective randomized trial comparing EC with SC for polyp detection has been published by our study group. In that study, a higher polyp detection rate for EC has been shown. However, that study had methodological limitations, such as a limited number of participating centers [9]. Moreover, withdrawal times were not measured. Thus, our study aimed to prospectively compare EC-assisted colonoscopy with standard colonoscopy for validating our initial study results in a broad multicenter setting. We hypothesized that the adenoma detection rate, with the use of Endocuff, is superior in comparison to standard colonoscopy.

## Materials and Methods

### Study Design

We performed a prospective randomized controlled multicenter trial, conducted at four academic endoscopy units in Germany (HELIOS Medical Centers Northeim, Siegburg, Helmstedt and the Department of Gastroenterology at University Medical Center Göttingen), comparing EC and SC for colonic adenoma detection. The protocol for this trial and supporting CONSORT checklist are available as supporting information; see Checklist S1 and Protocol S1. The study protocol was approved by the local ethics committees (approval-number 10/10/13, ethics commission of the University of Göttingen, approval date January 3, 2014; approval-number 2014039, ethics commission of the Medical Association Nordrhein, approval date March 11, 2014) and registered in a clinical trial database (http://ClinicalTrials.gov Identifier: **NCT02034929**). The present trial was registered before recruiting the first participant. The authors confirm that all ongoing and related trials for this study are registered.

### Patients

**Figure 1:**
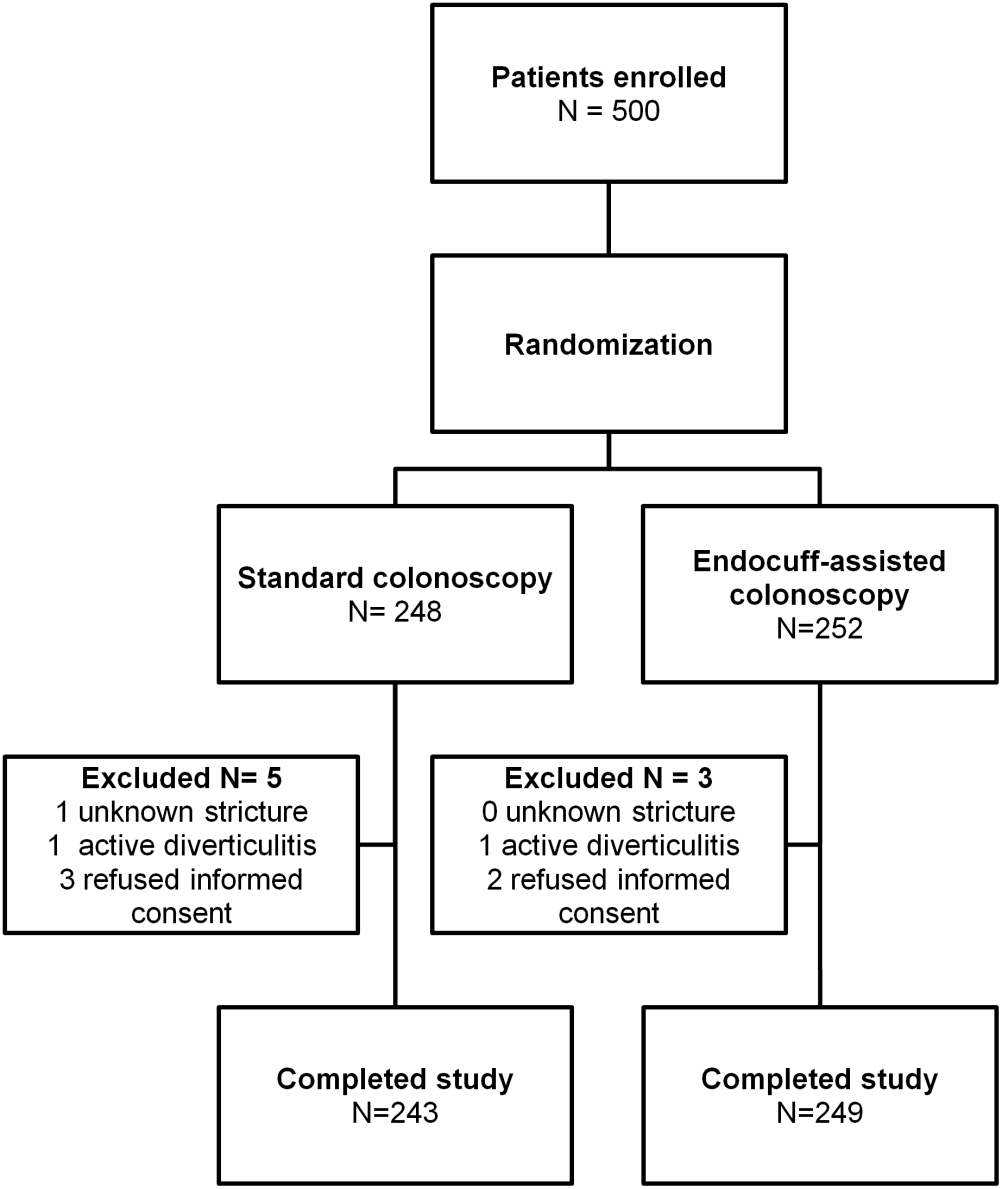
Enrollment flow chart.

A total of 818 consecutive patients underwent colonoscopy between February 2014 and July 2014, of whom 500 patients fulfilling the inclusion criteria were prospectively enrolled. After giving written informed consent, all patients underwent general physical examination and laboratory testing. Within a time interval of two weeks after enrollment, patients underwent either EC-assisted colonoscopy or standard colonoscopy. Block randomization (block size: 50) of the two groups (EC and SC) was performed by using computerized randomization lists. An independent researcher, who was not part of the endoscopy team, generated the allocation sequence and assigned participants to the groups. Participants were eligible for the study if they were at least 18 years of age and were scheduled for screening, surveillance or diagnostic colonoscopy (anemia or abdominal discomfort). Patients with known colonic strictures, status post partial colonic resection, acute diverticulitis within six weeks before the examination, acute exacerbation of chronic inflammatory bowel disease, pregnancy or inability to give informed consent were excluded from the study. The enrollment flow chart is shown in **Figure 1**. Clinical baseline data of patients enrolled is presented in **Table 1**.

**Table 1:** Baseline demographic and clinical characteristics.

Variable	EC	SC	p-Value
Patients, N	249	243	
Median age (years) [IQR]	64 [54–73]	63 [53–73]	0.572
Gender (male/female), N	122/127	109/134	0.358
First time colonoscopy, N (%)	85 (34)	92 (38)	0.390
Diabetes, N (%)	11 (4)	7 (3)	0.364
ASS medication, N (%)	28 (11)	33 (14)	0.432
Prior abdominal surgery, N (%)	42 (17)	44 (18)	0.718

ASS = acetylsalicylate; IQR = interquartile range.

### Endoscopic Procedures

#### Technical aspects

Colonoscopy using the EC device (Endocuff AEC120 or AEC140, Arc Medical, Leeds, UK) was performed with the device mounted on the tip of the endoscope. For the Fujifilm endoscopes (EC-590 WM4/WL4, Fujifilm Europe, Düsseldorf, Germany), we used the AEC120 device, for the Olympus colonoscopes (CF-H180AI/AL, Olympus, Tokyo, Japan), the AEC140 device was attached at the tip of the scope.

#### Definitions

Procedure time was defined as: beginning with the insertion of the colonoscope, including therapeutic interventions, and ending with removal of the endoscope.

Withdrawal time was defined as: beginning with withdrawal of the colonoscope from the caecal pole minus time spent for interventions, and ending with removal of the endoscope.

#### Procedures

Colon cleansing was achieved by using Moviprep (Norgine, Amsterdam, Netherlands) according to a standard bowel preparation protocol provided by the manufacturer. For sedation purposes, propofol was applied at an initial dose of 60 to 80 mg and repeated at fractionated doses of 40 to 80 mg, on demand, during the examination. If necessary, fentanyl at 50 to 100 µg for analgesia was intravenously administered. The colonoscopy started with the patient lying in the left lateral position and the colonoscope with or without the EC was pushed straight forward to the caecal pole. Routinely, 3 to 5 attempts for intubating the terminal ileum were made. A microchronometer was used to determine the overall procedure time and withdrawal time. Every detected polyp during withdrawal which was removed by standard forceps or snare, was put into a separate pot for histological analysis and individual assignment to the particular colonic location. The colonoscopies were performed by 10 highly experienced board-certified gastroenterologists, each of which having performed at least 3000 colonoscopies. All endoscopists had experience with at least 5 EC-assisted colonoscopies before participating in the study.

### Histopathology

The specimens were evaluated by three institutes of pathology. All lesions were classified as hyperplastic polyps, or serrated, tubular, tubulovillous, villous adenomas or carcinoma. A five-tiered classification and grading system according to the Vienna classification [10] (negative for dysplasia, indefinite for dysplasia, non-invasive low grade dysplasia [LGIN], non-invasive high grade dysplasia [HGIN] and invasive carcinoma) was used. All pathologists were blinded as to the method used for detection for all specimens.

### Study Outcome Measures

The primary outcome measure of the present study was direct comparison of EC-assisted colonoscopy with standard colonoscopy with regard to the adenoma detection rate (defined as the number of patients in whom at least one adenoma could be found). Secondary outcome measures included the polyp detection rate, number of polyps detected per colonoscopy, number of adenomas detected per colonoscopy, analysis of polyp distribution and polyp morphology in the different parts of the colon (rectum, sigmoid, descending colon, left flexure, transverse colon, right flexure, ascending colon and caecum). Secondary outcomes also included polyp detection proved by histology (hyperplastic polyp, LGIN, HGIN, invasive carcinoma), rate of caecal intubation, intubation rates of the ileum, overall colon cleansing level (graded as either poor, fair or good, according to the criteria of Leighton and colleagues) [11], overall procedure time, withdrawal time, and adverse events (severe bleeding, mucosal lacerations, perforation, cuff loss). The polyp size was estimated by comparison with open standard forceps (7 mm).

### Statistical Analysis and Sample Size Calculation

In modification of the statistical methods of Rastogi [3], the primary analysis compared the adenoma detection rate between the EC group and the SC group. It was assumed that the proportion of patients with at least one adenoma would be 20% [2] with SC and 32% with EC-assisted colonoscopy. A priori analysis with Pearson’s chi-squared test for two proportions was performed. A minimum of 225 patients per group were required to achieve at least an 80% power in order to detect a 12% difference in the ADR. A type I error rate of 5%, using two-sided tests was used. Statistical power analysis was performed using G*Power 3.17 [12]. A Forest plot was created using Forest Plot Viewer (SRA Int., Inc.). All other analyses were performed using SPSS 17.0 (Chicago, II, USA). Quantitative variables were expressed as median (interquartile range), while categorical variables were presented as total numbers and percentages. A two-tailed chi-squared test for categorical variables and a Wilcoxon-Mann-Whitney test for quantitative variables were applied. A p-value less than 0.05 was considered statistically significant. Multivariate binary logistic regression analysis with backward elimination for identifying possible predictors of adenoma detection was performed and presented as a Forest plot. All statistical analyses were supervised by the Institute of Biostatistics and Clinical Research of the University of Münster, Germany.

## Results

### Patient Characteristics

We prospectively enrolled a total of 500 patients into the study who fulfilled the inclusion criteria. 8 patients were excluded, either because of study withdrawal, stenosis or active inflammation (Enrollment flow chart see **Figure 1**). Finally, a total of 492 patients completed the study. The median age of the study cohort was 64 years [IQR 54–73], with no statistical differences between the two groups in terms of gender, first time colonoscopy, diabetes, prior abdominal surgery or aspirin medication (EC vs. SC, **Table 1**).

### Procedural Characteristics

The caecum could be reached in almost all patients, regardless of whether or not the EC was used (EC: 96%[95%CI 93–98%] vs. SC: 94%[95%CI 91–97%]; p = 0.624). The main procedural outcomes are presented in **Table 2**. There were no statistical differences in terms of ileum intubation rates (EC: 66%[95%CI 60–72%] vs. SC 71%[95%CI 65–77%], p = 0.239), median overall procedural times, median withdrawal times and bowel preparation results. The amount of propofol applied during the examinations was slightly higher in the SC group (150 mg [IQR 120–200] vs. 170 mg [130–210], p = 0.004).

**Table 2:** Main outcomes of endoscopic procedures.

Variable	EC	SC	p-Value
Cecum intubation, N (%)	238 (96)	229 (94)	0.624
Ileum intubation, N (%)	165 (66)	173 (71)	0.239
Procedure time (min), median [IQR]	17 [13]–[21]	17 [14]–[20]	0.959
Withdrawal time (min), median [IQR]	6.32 [5.5–8.0]	6.05 [5.5–8.0]	0.524
Cleanliness score, median [IQR]	1 [1]–[2]	1 [1]–[2]	0.797
1 = good, N (%)	185 (74)	176 (72)	
2 = fair, N (%)	53 (21)	54 (22)	
3 = poor, N (%)	12 (5)	12 (5)	
Propofol dosage (mg), median (IQR)	150 [100–200]	170 [130–210]	**0.004**

IQR: interquartile range.

### Polyp Detection Rate

A total of 501 polyps were detected during colonscopy within the study period: In the EC-group, 312 polyps were found while in the SC-group 189 polyps could be retrieved. The polyp detection rate (PDR), that is the number of patients in whom at least one polyp was detected, was significantly higher in the EC-group compared to the SC-group (55.4%[95%CI 49–62%] vs. 38.4%[95%CI 32–44%], p<0.0001) resulting in a PDR increase of 17% (95%CI 6.4–23.8%). Significantly more polyps smaller than 1 cm in size as well as more sessile polyps were detected in the EC group (N = 272 vs. 172, p<0.0001 and N = 250 vs. 145, p<0.0001 for sessile polyps). The detection rates for flat or pedunculated polyps were not different. For details see **Table 3**.

**Table 3:** Polyp and adenoma detection analysis.

	EC#	SC#	p-Value
Polyp detection rate, N (%)	138 (55.4)	93 (38.4)	**<0.0001**
Polyps per patient, median [IQR]*	2 [1]–[3]	1 [1]–[3]	0.250
Polyp by size analysis, N (%)			
≥1 cm	40 (12.8)	17 (9)	**0.005**
<1 cm	272 (87.2)	172 (91)	**<0.0001**
Polyp by morphology analysis, N (%)			
Sessile	250 (80.1)	145 (76.7)	**<0.0001**
Flat	48 (15.4)	33 (17.4)	0.072
Pedunculated	14 (4.5)	11 (5.9)	0.275
Adenoma detection rate (ADR), N (%)	87 (35.4)	50 (20.7)	**<0.0001**
Number of adenomas (LGIN), N (%)	138 (93)	87 (99)	**0.002**
Number of adenomas (HGIN), N (%)	6 (4)	1 (1)	0.061
Number of carcinomas, N (%)	5 (3)	0 (0)	0.061
Adenomas per patient, median [IQR]*	1 [1]–[2]	1 [1]–[3]	0.851
Hyperplastic Polyp detection rate, N (%)	71 (28.7)	51 (21.2)	0.053
Hyperplastic polyps per patient, median [IQR]*	1 [0–2]	1 [0–1]	0.922

*only patients considered in whom at least one adenoma or polyp, respectively were detected.

# 3 polyps (EC) and two polyps (SC) not retrieved endoscopically.

### Distribution of Polyp Detection

When using the Endocuff, the sigmoid polyp detection rates (30.1%[95%CI 24–36%] vs. 17.3%[95%CI 12–22%], p = 0.001) and the ascending colon polyp detection rates (12.4%[95%CI 8–17%] vs. 5.8%[95%CI 3–7%], p = 0.010) were significantly higher. In all other colonic segments, no difference in polyp detection rates could be observed. For absolute number of polyps, significantly more polyps <1 cm in size could be detected in the sigmoid (108 vs. 52, p = 0.001) and ascending colon (31 vs. 14, p = 0.036) with the use of EC. When analyzing the polyp morphology, EC-assisted colonoscopy detected significantly more sessile polyps in the sigmoid (99 vs. 45, p = 0.002) and caecal region (29 vs. 7, p = 0.003) as well as significantly more flat polyps in the transverse colon (10 vs. 0, p = 0.015) compared to SC. Details are presented in **Tables 3** **and** **4**.

**Table 4:** Colonic distribution, size and morphology of polyps.

Variable, N	EC	SC	P-Value
Rectum polyp detection rate, N (%)	40 (16)	31 (12.8)	0.297
No of Rectum polyps <1 cm, N (%)	52 (90)	45 (96)	0.544
No of Rectum polyps >1 cm, N (%)	6 (10)	2 (4)	0.165
sessile, N (%)	47 (81)	35 (74)	0.279
flat, N (%)	8 (14)	7 (15)	0.549
pedunculated, N (%)	3 (5)	5 (11)	0.455
Sigmoid polyp detection rate, N (%)	75 (30.1)	41 (17.3)	**0.001**
No of Sigmoid polyps <1 cm, N (%)	108 (91)	52 (95)	**0.001**
No of Sigmoid polyps >1 cm, N (%)	11 (9)	3 (5)	0.053
sessile, N (%)	99 (83)	45 (82)	**0.002**
flat, N (%)	12 (10)	7 (13)	0.118
pedunculated, N (%)	8 (7)	3 (5)	0.214
Descending colon polyp detection rate, N (%)	14 (6)	18 (7)	0.422
No of descending colon polyps <1 cm, N (%)	13 (87)	15 (75)	0.782
No of descending colon polyps >1 cm, N (%)	2 (13)	5 (25)	0.241
sessile, N (%)	11 (73)	15 (75)	0.371
flat, N (%)	3 (20)	4 (20)	0.972
pedunculated, N (%)	1 (7)	1 (5)	0.986
Left flexure polyp detection rate, N (%)	4 (1.6)	5 (2)	0.709
No of left flexure polyps <1 cm, N (%)	3 (75)	6 (100)	0.456
No of left flexure polyps >1 cm, N (%)	1 (25)	0 (0)	0.324
sessile, N (%)	3 (75)	5 (83)	0.676
flat, N (%)	1 (25)	1 (17)	0.986
pedunculated, N (%)	0 (0)	0 (0)	1.000
Transverse colon detection rate, N (%)	23 (9.2)	15 (6.2)	0.203
No of transversum polyps <1 cm, N (%)	24 (80)	16 (94)	0.405
No of transversum polyps >1 cm, N (%)	6 (20)	1 (6)	0.107
sessile, N (%)	19 (63)	17 (100)	0.900
flat, N (%)	10 (33)	0 (0)	**0.015**
pedunculated, N (%)	1 (4)	0 (0)	0.323
Right flexure polyp detection rate, N (%)	13 (5)	10 (4)	0.561
No of right flexure polyps <1 cm, N (%)	15 (94)	11 (92)	0.159
No of right flexure polyps >1 cm, N (%)	1 (6)	1 (8)	0.986
sessile, N (%)	13 (81)	11 (92)	0.301
flat, N (%)	3 (19)	1 (8)	0.328
pedunculated, N (%)	0 (0)	0 (0)	1.000
Ascending colon polyp detection rate, N (%)	31 (12.4)	14 (5.8)	**0.010**
No of ascendens polyps <1 cm, N (%)	31 (84)	14 (88)	**0.036**
No of ascendens polyps >1 cm, N (%)	6 (16)	2 (12)	0.166
sessile, N (%)	29 (78)	11 (69)	0.015
flat, N (%)	7 (19)	3 (19)	0.329
pedunculated, N (%)	1 (3)	2 (12)	0.984
Caecum polyp detection rate, N (%)	24 (9.6)	14 (5.8)	0.107
No of caecum polyps <1 cm, N (%)	26 (79)	13 (81)	0.110
No of caecum polyps >1 cm, N (%)	7 (21)	3 (19)	0.221
sessile, N (%)	29 (88)	7 (44)	**0.003**
flat, N (%)	4 (12)	9 (56)	0.335
pedunculated, N (%)	0 (0)	0 (0)	1.000

No = number; bold = significant differences.

### Adenoma Detection Rate (ADR)

The adenoma detection rate (meaning all patients in whom at least one adenoma was detected during colonoscopy) increased by 14.7%[95%CI 6.9–22.5%] when using the Endocuff. In the EC group, the ADR was 35.4%[95%CI 29–41%] compared to 20.7%[95%CI 15–26%] in the SC group (p<0.0001) (**Table 3**). In total, 237 neoplastic lesions were found within the study period, with significantly more LGIN in the EC group (N = 138 vs. 87, p = 0.002). The number of HGIN and carcinomas detected by EC did not differ statistically compared to SC (HGIN: N = 6 vs. 1, p = 0.061; carcinoma: N = 5 vs. 0, p = 0.061) (**Table 3**). Histological polyp analysis was not possible in 5 polyps. These polyps could either not be retrieved or were left in situ due to anticoagulation treatment.

### Adverse Events

In the majority of colonoscopies, no adverse events occurred in either group. Significantly more minor mucosal lacerations without any clinical impact were observed in EC-assisted procedures in comparison to SC (EC: 18 vs. SC: 2, p<0.0001). No major bleeding events, perforations or cuff losses occurred (**Table 5**).

**Table 5:** Procedural adverse events.

Adverse Event	EC	SC	p-Value
Minor mucosal lacerations, N (%)	18 (7.3)	2 (0.8)	**<0.0001**
Major bleeding	0	0	–––––
Perforation	0	0	––––
Loss of Cuff	0	0	––––
SpO_2_ decline (<90%)	0	0	––––

SpO_2_ = Saturation of peripheral Oxygen; bold = significant differences.

### Independent Predictors of Colonic Adenoma Detection

**Figure 2:**
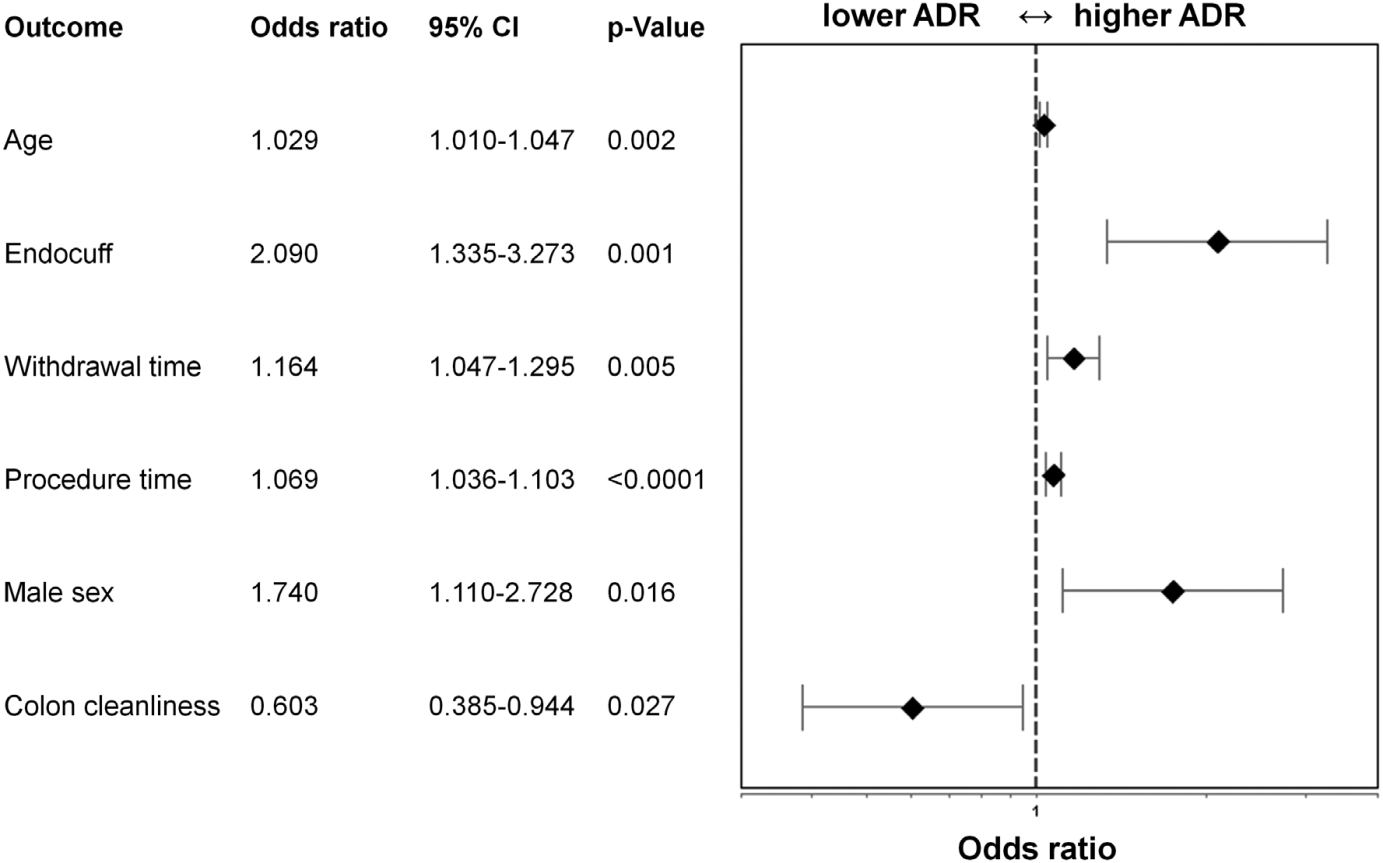
Forest plot showing the results of multivariate regression analysis for adenoma detection. The x-axis represents the Odds ratio on a log scale with the reference line (dashed), Odds ratios (diamond) and 95% CI (whiskers).

Patients, in whom at least one adenoma was detected, were significantly older compared to those without adenoma detection (median age 69 [61–75] vs. 63 [52–73], p = <0.0001). In the adenoma detection cohort, the proportion of male patients was significantly higher (58%[95%CI 50–67%] vs. 42%[95%CI 37–47%], p = 0.001). Moreover, the withdrawal times and overall procedure times were significantly longer in those patients where adenomas were detected (withdrawal time 7 [6.30–8.25] vs. 6 [5.09–7.00] min, p<0.0001; procedure time: 20 [16]–[25] vs.15.5 [13]–[20], p<0.0001). We additionally performed multivariate logistic regression analysis and found age (OR 1.029, 95%CI 1.010–1.047, p = 0.002), male sex (OR 1.740, 95%CI 1.110–2.728,p = 0.016), withdrawal time (OR 1.164, 95%CI 1.047–1.295, p = 0.005), procedure time (OR 1.069, 95%CI 1.036–1.103, p<0.0001), colon cleanliness (OR 0.603, 95%CI 0.385–0.944, p = 0.027) and use of Endocuff (OR 2.090, 95%CI 1.335–3.273, p = 0.001) as independent predictors of adenoma detection (**Figure 2**).

## Discussion

Colonoscopy performed within colon cancer prevention programs remains the gold standard for colorectal cancer screening. The adenoma detection rate (ADR) seems to be most crucial in preventing interval colon cancer, as a suboptimal ADR is significantly associated with a higher incidence of colon cancer [2]. In a recently published study by Corley et al. with analysis of 314872 colonoscopies, the ADR was inversely associated with the risk of interval colon cancer. Each percent increase in ADR resulted in a 3 percent decrease of colon cancer risk (HR 0.97; 95%CI 0.96–0.98) [13]. An ADR of at least 20% is generally accepted as sufficient for colon cancer prevention programs and should be the standard for colon cancer prevention centers [2]. In our study, an ADR of 20.7% in the standard colonoscopy arm was achieved. We were therefore able to consider this cohort as a representative control group.

Another aspect is the importance of the withdrawal time for the ADR. Earlier studies have shown, that insufficient withdrawal times correlate with a lower ADR and thus with a higher incidence of colorectal cancer [14], [15]. In our study, we found the withdrawal time to be an independent predictor for adenoma detection in multivariate analysis, confirming previous study results. Moreover, in our study, the median withdrawal time routinely exceeded 6 minutes in each group, which is considered sufficient for standard colonoscopy according to current guidelines [14], [16], thus excluding bias due to insufficient withdrawal times. Our study showed that Endocuff-assisted colonoscopy significantly increased the ADR by 14.7% compared to SC. Superiority of EC over SC could especially be seen in anatomic obstacles like the sigmoid.

Other technical innovations like cap-assisted colonoscopy, back-to-back colonoscopy or narrow band imaging (NBI) or a combination of several methods were evaluated for higher polyp and adenoma detection rates [17]–[21]. All of these procedures have shown more or less benefit compared to standard colonoscopy. However, all future procedures with the goal of improving colonic adenoma detection need to have broad acceptance, especially in terms of procedure time and expense.

It holds true that back-to-back colonoscopy improves polyp and adenoma detection rates [20]. However, it is self evident that performing a colonoscopy twice is no option for broad endoscopic colon cancer prevention programs. Moreover, there would be serious ethical considerations when exposing the patient to the risk of a double colonoscopy.

The value of NBI or FICE techniques in the colon is critically discussed, since not all studies have shown improvement of the ADR. In a recently published randomized trial comparing NBI with high definition colonoscopy for the detection of adenomas, NBI was not able to improve the ADR [22]. Neither NBI nor FICE increased the ADR in a randomized tandem trial conducted by Chung [17].

Water-immersion or water-exchange colonoscopy is a new emerging technique which is still under evaluation. However, in a recently published meta-analysis comparing water immersion and water exchange with air insufflation colonoscopy, a reduction in procedural pain could be observed, but no difference in adenoma detection [23].

The effort and the aspect of losing water through the anus makes its use doubtful for broad colon cancer prevention programs.

Cap-assisted colonoscopy (CAC) showed conflicting results for improving ADR. While in the study by Rastogi et al., ADR improvement was observed [3], a recently published two-center trial conducted by de Wijkersooth et al. failed to show an improvement in ADR [24]. Nevertheless, this technique seems feasible and the advantage of CAC becomes clear, especially in the right colonic flexure [25]. However, more training is likely to be necessary as compared to the endocuff due to the technical nature of CAC and its effect on the view of the examiner. A prospective randomized head-to-head comparison of CAC with EC-assisted colonoscopy is not yet available, but would be of great value.

In another recently published randomized controlled trial, conducted by Gralnek et al., the adenoma miss rate was significantly lower with the use of a novel full-spectrum endoscopy platform as compared to standard colonoscopy [21]. However, complex technical investment is necessary, making broad use in screening programs difficult.

A great strength of our study is its multicenter randomized character and its statistical power. So far, this is the study with the largest number of patients included. We further wanted to determine, which independent factors contribute significantly to the variability of the ADR. Therefore we performed multivariate binary logistic regression analysis. Variations of multivariate regression include forward selection and backward elimination methods. We decided to choose backward elimination because all independent factors begin in the model and non-influential variables are eliminated. In our multivariate analysis all included variables remained in the model. We proved the Endocuff, for the first time, to be an independent factor for adenoma detection (OR 2.09). We also found a significantly higher proportion of male patients in whom at least one adenoma was found (OR 1.74). This observation is consistent with previously published data [26]–[28]. An important co-finding was, that ileal intubation rates were similar in both groups with no statistically significant difference.

The overall procedure time with EC did not differ statistically compared to standard colonoscopy.

Statistical differences were seen in the polyp detection rates for polyps less than 1 cm in diameter and to a lesser extent for those larger than 1 cm. Obviously, even larger polyps can better be detected during EC-assisted colonoscopy in critical anatomical positions. The polyp detection rate in the sigmoid region was notably higher with use of EC. Thus, the benefit of the Endocuff is particularily favourable as the majority of polyps occure in the left hemicolon^29^.

According to the inverse correlation of ADR and interval colon cancer risk (one percent increase of ADR results in three percent reduction of colon cancer risk)^13^, our observed increase of 14.7% in the ADR does consequently have the potential to reduce the interval colon cancer risk by more than 40%. In our study, for every seven patients screened by EC assisted colonoscopy an additional adenoma could be detected (number needed to screen (NNS = 1/(ADR_EC_−ADR_SC_)). Assuming a 2 to 5% risk for adenoma to carcinoma progression within the following 10 years, 140 to 350 EC-assisted colonoscopies would be necessary to prevent one colon cancer case. Hence, the use of EC is economically reasonable.

In our study, no significant adverse effects were detected, besides minor mucosal lacerations without any clinical impact. We have not noticed any Endocuff losses during withdrawal as previously reported by our study group [9]. It is likely that technical advances of the Endocuff with firmer attachment to the tip of the endoscope have prevented this adverse event.

### Conclusions

The use of EC is safe, feasible and significantly improves the adenoma detection rate. As previously discussed, improving the ADR correlates with a decrease in colon cancer risk. Future follow-up studies are necessary to show the impact of EC assisted colonoscopy on colon cancer morbidity and mortality, before broad use of the EC device in colon cancer prevention programs can be recommended.

There has been further development of the Endocuff device with even longer arms (Endocuff vision) that have the potential to improve ADR. The first pilot study on this device showed promising results [29].

### Limitations

The impact of our findings on colon cancer mortality prevention remains uncertain, thus follow-up studies are necessary.

Due to technical reasons such as the occasional visibility of the rubber arms of the endocuff and the small, but detectable resistence during the endoscopy proceding into the colon, this study was not blinded.

## Supporting Information

Checklist S1 **Consort checklist**
(DOC)

Protocol S1 **Study protocol**
(DOCX)
