# Supplementary material for: Higher Adenoma Detection Rates with Endocuff-Assisted Colonoscopy – A Randomized Controlled Multicenter Trial
Source: PLoS One. 2014 Dec 3;9(12):e114267. doi: 10.1371/journal.pone.0114267 (PMC4255000; doi:10.1371/journal.pone.0114267)
Supplement: Protocol S1 — Study protocol (DOCX) [file pone.0114267.s002.docx]

**Study protocol Endocuff**

This study protocol was submitted to and approved by the ethics committee of the University of Göttingen before the trial began on January 3, 2014

Study title: Endocuff-assisted vs. standard colonoscopy for detection of adenomas

Short title: Adenoma detection rate of Endocuff

Study director NOM: Priv.-Doz. Dr. med. Tobias Meister

Deputy study director NOM: Dr. med. Martin Floer

Medizinische Klinik II

Helios Albert-Schweitzer-Klinik Northeim

Tel.: 05551/97-1850

Study director HE: Prof. Dr. med. Detlev Ameis

Medizinische Klinik II

Helios St. Marienberg Klink Helmstedt

Conringstraße 26, 38350 Helmstedt

Telefon: 0 53 51-14 - 0

Study director UMG: Prof. Dr. med. Volker Ellenrieder

Klinik für Gastroenterologie II

UMG

Study director Siegburg Prof Dr. med. Michael Schepke

Medizinische Klinik

Helios-Klinikum Siegburg

Tel.: 02241-18-2226

**II. Synopsis Endocuff Study**

Study title: Endocuff-assisted vs. standard colonoscopy for detection of adenomas

Study indication: Determination of the adenoma detection rate

Estimated duration of study: Estimated start of recruiting period 01.2013

End of recruiting period 01.07.2014

End of follow up: -

End of Study (including analysis 01 .10.2014)

Study goals:

Primary study goal: Comparison of standard colonoscopy with/ without Endocuff in relation to registration of at least one adenoma per colonoscopy (adenoma detection rate)

Secondary study goal: 1. Bowel preparation results

2. Registration of complications

3. Detection of neoplasms in regard of localization, number, size,

morphology

4. Caecum intubation time

5. Procedure time

6. Withdrawal time

Measured parameters: Localization of neoplasms (by means of data collection sheet)

Number of neoplasms (by means of data collection sheet)

Size of neoplasms (by means of data collection sheet)

Morphology of polyps (by means of data collection sheet)

Time measurement

Study design: Multicenter, prospective, randomized, not placebo-controlled diagnosic study

Study participants: Planned number: 225 patients with endocuff, 225 patient without endocuff

Study centers: Albert-Schweitzer-Klinik Northeim, Germany

Helios-Klinikum Siegburg, Germany

Universitätsmedizin Göttingen, Germany

Helios Klinikum Helmstedt, Germany

Evaluated diagnostic

procedure: Colonoscopy

III. Study criteria:

- Inclusion criteria:
  - Colonoscopy independent from indication
  - Age ≥ 18
  - Patient is able to provide informed consent for the procedure
- Exclusion criteria:
  - IBD stenosis
  - Pregnant of nursing patient
  - Acute diverticulitis
  - Active IBD
  - Age ≤ 18
  - Coagulation disorder (INR>1.4 , Thrombocytes < 50000/µl)

IV. Study procedure

| Study period |  |  |
| --- | --- | --- |
| **Ward round** | V0 | V1 |
| **Day** | 1 | 2 |
| **Informed consent** | X |  |
| **Demographic data** | X |  |
| **Medical history** | X |  |
| **Inclusion / Exclusion criteria** | X |  |
| **Physical examination** | X |  |
| **Vital signs** | X | X |
| **Concomitant medication** | X |  |
| **Laboratory values** | X |  |
| **Randomization Endocuff/without Endocuff** |  | X |
| **Colonoscopy with/without Endocuff** |  | X |
| **Completion of the data collection sheet after the colonoscopy** |  | X |

Data collection sheet Number:

**Endocuff study**

Field 1

Center:

O Northeim O Siegburg O Göttingen O Helmstedt

Field 2

Patient data :

Age______________

Gender: O Female O Male

Previous operations of the abdomen: ___________________

First colonoscopy in his/her life: O Yes O No

Diabetes: O Yes O No

Polyposis: O Yes O No

IBD: O Yes O No

ASS Medication: O Yes O No

Field 3

O use of Endocuff

O without Endocuff

Field 4

Bowel preparation:

O Excellent (>90% of mucosa seen, mostly liquid colonic contents, minimal suctioning needed for adequate visualization)

O Good (>90% of mucosa seen, mostly liquid colonic contents, significant suctioning needed for adequate visualization)

O Fair (>90% of mucosa seen, mixture of liquid and semisolid colonic contents, which could be suctioned and/or washed )

O Inadequate (<90% of mucosa seen, mixture of semisolid and solid colonic contents, which could not be suctioned or washed)

Field 5

O Complete colonoscopy

- O with terminal ileum intubation

- O without terminal ileum intubation

O Partial colonoscopy (until ____cm from anus)

Field 6

O **No** polyps detected during procedure

O Polyps detected during procedure

**Location/ size / number of polyps**

Cecum

O Polyp Size <1cm, Number __ O Polyp size >1cm, Number __

Ascending colon

O Polyp Size <1cm, Number __ O Polyp size >1cm, Number __

Hepatic flexure (right)

O Polyp Size <1cm, Number __ O Polyp size >1cm, Number __

Transvers colon

O Polyp Size <1cm, Number __ O Polyp size >1cm, Number __

Splenic flexure (left)

O Polyp Size <1cm, Number __ O Polyp size >1cm, Number __

Descending colon

O Polyp Size <1cm, Number __ O Polyp size >1cm, Number __

Sigmoid colon

O Polyp Size <1cm, Number __ O Polyp size >1cm, Number __

Rectum

O Polyp Size <1cm, Number __ O Polyp size >1cm, Number __

Field 7

**Location/ size/ number of polyps according to histologic etiology**

Cecum

O Adenoma, number __ O LGIN, number__ O HGIN, number __ O Hyperplastic, number __

O Cancerous, number __

Ascending colon

O Adenoma, number __ O LGIN, number__ O HGIN, number __ O Hyperplastic, number __

O Cancerous, number __

Hepatic flexure (right)

O Adenoma, number __ O LGIN, number__ O HGIN, number __ O Hyperplastic, number __

O Cancerous, number __

Transverse colon

O Adenoma, number __ O LGIN, number__ O HGIN, number __ O Hyperplastic, number __

O Cancerous, number __

Splenic flexure (left)

O Adenoma, number __ O LGIN, number__ O HGIN, number __ O Hyperplastic, number __

O Cancerous, number __

Descending colon

O Adenoma, number __ O LGIN, number__ O HGIN, number __ O Hyperplastic, number __

O Cancerous, number __

Sigmoid colon

O Adenoma, number __ O LGIN, number__ O HGIN, number __ O Hyperplastic, number __

O Cancerous, number __

Rectum

O Adenoma, number __ O LGIN, number__ O HGIN, number __ O Hyperplastic, number __

O Cancerous, number __

Field 8

Polyp - Morphology

Cecum

O Polyp sessile number ___, O Polyp flat number __ O Polyp pedunculated number __

Ascending colon

O Polyp sessile number ___, O Polyp flat number __ O Polyp pedunculated number __

Hepatic flexure (right)

O Polyp sessile number ___, O Polyp flat number __ O Polyp pedunculated number __

Transverse colon

O Polyp sessile number ___, O Polyp flat number __ O Polyp pedunculated number __

Splenic flexure (left)

O Polyp sessile number ___, O Polyp flat number __ O Polyp pedunculated number __

Descending colon

O Polyp sessile number ___, O Polyp flat number __ O Polyp pedunculated number __

Sigmoid colon

O Polyp sessile number ___, O Polyp flat number __ O Polyp pedunculated number __

Rectum

O Polyp sessile number ___, O Polyp flat number __ O Polyp pedunculated number __

Field 9

Procedure time (min): _____

Withdrawal time (min) (stopwatch is paused for procedures like: suction, biopsy, polypectomy): ___

Sedation necessity: Propofol (mg) ____ ; Opiates (Morphium- equivalents) (mg) _______

Field 10

Complications

O none

O yes O perforation, O mucosa-laceration bleeding, O bleeding, O loss of Endocuff
